# Comparison of Acute Gastrointestinal Toxicity of Intensity-Modulated Radiotherapy Versus Three-Dimensional Conformal Radiotherapy in Patients of Carcinoma Cervix

**DOI:** 10.7759/cureus.48876

**Published:** 2023-11-16

**Authors:** Sanjukta Padhi, Bikash Ranjan Mahapatra, Kartik Chandra Pati, Bijayalaxmi Sahoo, Satyabrata Kanungo, Tanushree Mishra, Anupam Muraleedharan

**Affiliations:** 1 Radiation Oncology, Acharya Harihar Post Graduate Institute of Cancer, Cuttack, IND; 2 Radiation Oncology, Utkal Hospital, Bhubaneswar, IND; 3 Community Medicine, Fakir Mohan Medical College and Hospital, Balasore, IND; 4 Radiation Oncology, Kalinga Institute of Medical Sciences, Bhubaneswar, IND; 5 Radiation Oncology, Postgraduate Institute of Medical Education & Research (PGIMER) and Capital Hospital, Bhubaneswar, IND; 6 Radiation Oncology, ESI Hospital, Bhubaneswar, IND; 7 Radiation Oncology, All India Institute of Medical Sciences, Bhubaneswar, IND

**Keywords:** cervical cancer, acute bowel toxicity, imrt, 3dcrt, external beam radiotherapy

## Abstract

Introduction

Cervical cancer is the most common gynaecological malignancy worldwide, with a higher prevalence in middle- and low-income countries. Chemoradiotherapy, followed by intracavitary brachytherapy, is the treatment of choice in locally advanced cervical cancer. The most common acute side effect of external beam radiotherapy (EBRT) is bowel toxicity in the form of diarrhoea and abdominal cramps. The treatment techniques of EBRT were revolutionised with the advent of intensity modulation. This study aims to prospectively analyse whether the dosimetric advantage of intensity-modulated radiotherapy (IMRT) over three-dimensional conformal radiotherapy (3DCRT) is translated clinically into a decrease in acute toxicity.

Method

Twenty-four patients were randomised into two groups: the 3DCRT and the IMRT. Acute gastrointestinal (GI) toxicity was assessed during treatment using radiation therapy oncology group grading. The factors under consideration were age, stage of the disease, treatment technique, chemotherapy, and the intention of therapy (radical or adjuvant). The mean bowel bag dose of the two techniques was analysed.

Result

Among the factors under consideration, it was found that the treatment technique was the only factor that had a significant association with acute bowel toxicity in both univariate (p = 0.036) and multivariate analyses (p = 0.028). The mean V25 (the volume receiving 25 Gy), V45, and V50 of the bowel bag in the IMRT arm were significantly less than the 3DCRT arm. Grades 2 and 3 acute bowel toxicities were also higher in the 3DCRT arm.

Conclusion

The treatment technique is essential to determining acute GI toxicity during pelvic radiotherapy. With IMRT, the dose to the bowel bag and, in turn, the acute bowel toxicity can be reduced.

## Introduction

Globally, cervical malignancy is one of the leading gynaecological malignancies, with an incidence of 0.6 million (3.1% of all newly diagnosed cancers) [1]. This is the second most common female malignancy after breast cancer in India. India also adds 20% to the global cervical cancer burden [2].

The management of cervical cancer depends on the stage of the disease, the desire for fertility, comorbidities, and overall patient preference. Surgery and radiotherapy are treatment options for early-stage cervical cancer [3,4]. Whereas, for locally advanced disease, concurrent chemoradiation followed by brachytherapy is the treatment of choice [5-10]. Adjuvant therapy depends upon the presence of risk factors like margin status, lymph node positivity, parametrial involvement, bulky disease (>4cm), lymphovascular space invasion, or deep stromal invasion [5,11].

The delivery of radiation comes with its risks and benefits. This is attributed mainly to the dose received by normal tissues around the tumour, resulting in radiation-induced toxicity. Gastrointestinal (GI) complications are a significant concern. The bowel tolerance is significantly less than that for tumour killing [12].

Over the decades, radiotherapy techniques have evolved in conformity and normal tissue sparing. Intensity-modulated radiotherapy (IMRT) can achieve greater conformity by optimally modulating the intensity of individual beams, thereby reducing the toxicities.

This study attempts to analyse dosimetry and the incidence of acute GI toxicity in patients treated with IMRT and three-dimensional conformal radiotherapy (3DCRT) for cervical cancer. This study also aims to determine whether the higher conformality guaranteed by IMRT translates into clinical benefits.

## Materials and methods

This prospective randomised study was conducted at an Eastern India tertiary care cancer centre from April 2017 to October 2018. The study population was patients of cervical cancer of the International Federation of Gynaecology and Obstetrics (FIGO) stages IB2 to IVA who required radical radiotherapy or concurrent chemoradiotherapy and post-operative cervical cancer with indications for adjuvant radiotherapy or concurrent chemoradiotherapy [13]. The inclusion criteria for this study were histologically confirmed cervical cancer, age >18 years, Eastern Oncology Cooperative Group (ECOG) performance status <2, and no prior history of chemotherapy and radiotherapy. The patients who needed extended field irradiation because of para-aortic lymphadenopathy, those with collagen vascular diseases, and pregnant patients were excluded from this study. After getting institutional ethics committee clearance, the patients were recruited for the study. After a detailed history, thorough systemic and pelvic examinations were done. Per vaginal and per rectal examinations were done to assess the size and extent of the disease. The staging was done as per FIGO 2009 guidelines [13]. Every patient was investigated with complete blood count (CBC), serum urea and creatinine, X-ray chest, ultrasonography of the abdomen and pelvis, and echocardiography. After confirmation of diagnosis by histopathological examination, informed consent was taken from all the patients enrolled in the study.

**Figure 1:**
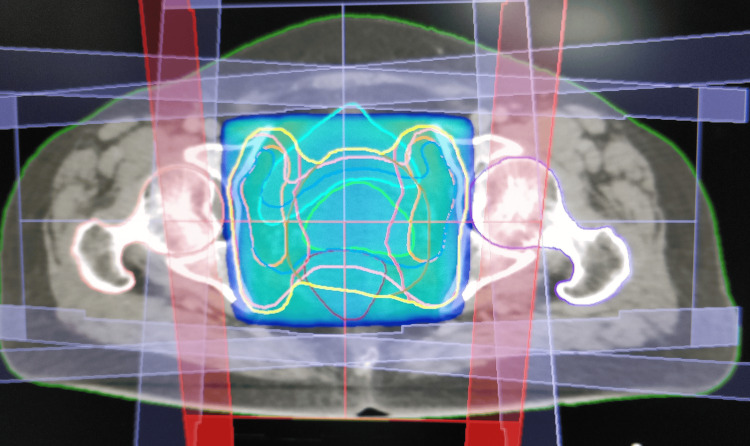
3DCRT plan showing the colour wash with 95% isodose coverage (axial section) 3DCRT, three-dimensional conformal radiotherapy

**Figure 2:**
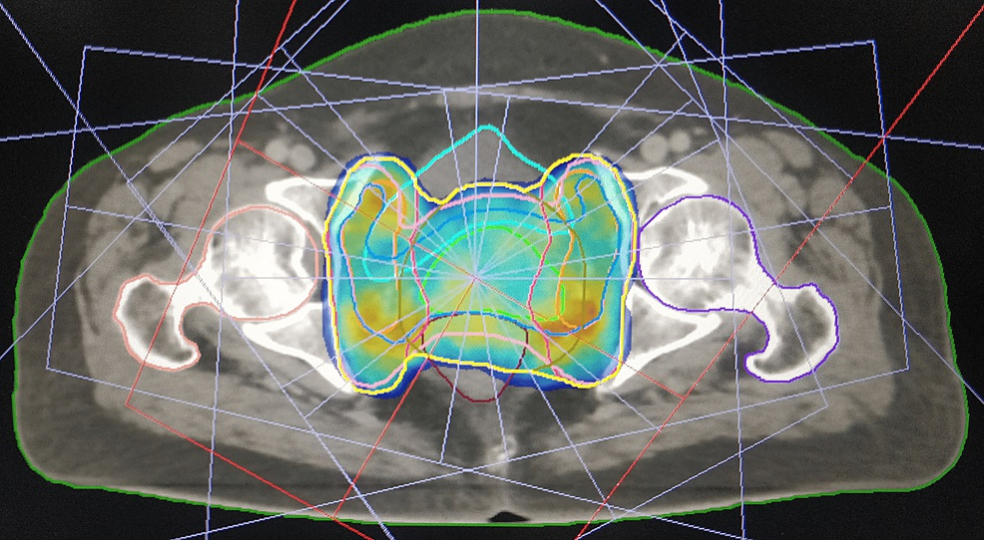
IMRT plan showing the colour wash with 95% isodose coverage (axial section) IMRT, intensity-modulated radiotherapy

**Figure 3:**
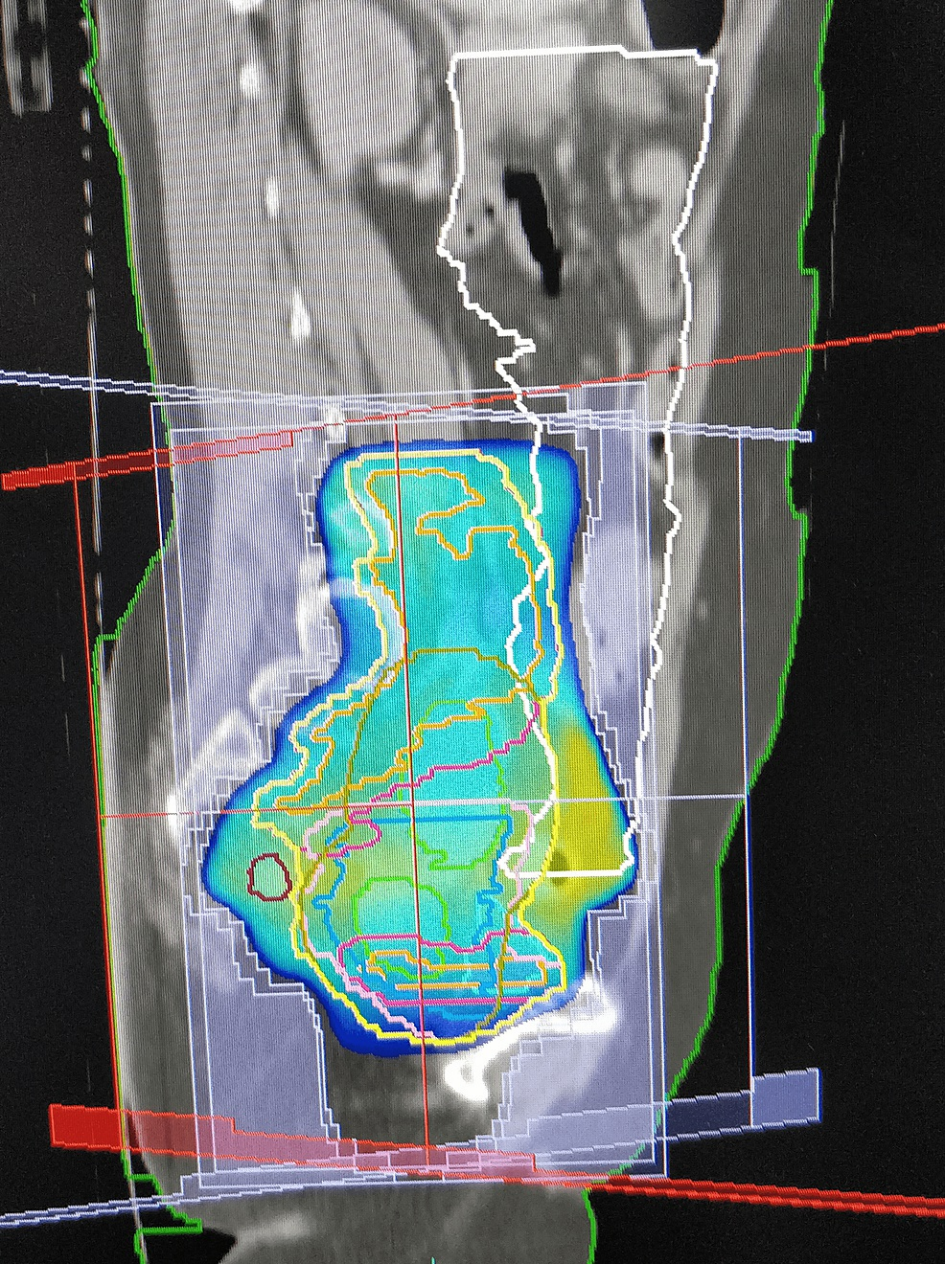
3DCRT plan showing the colour wash with 95% isodose coverage (sagittal section) 3DCRT, three-dimensional conformal radiotherapy

**Figure 4:**
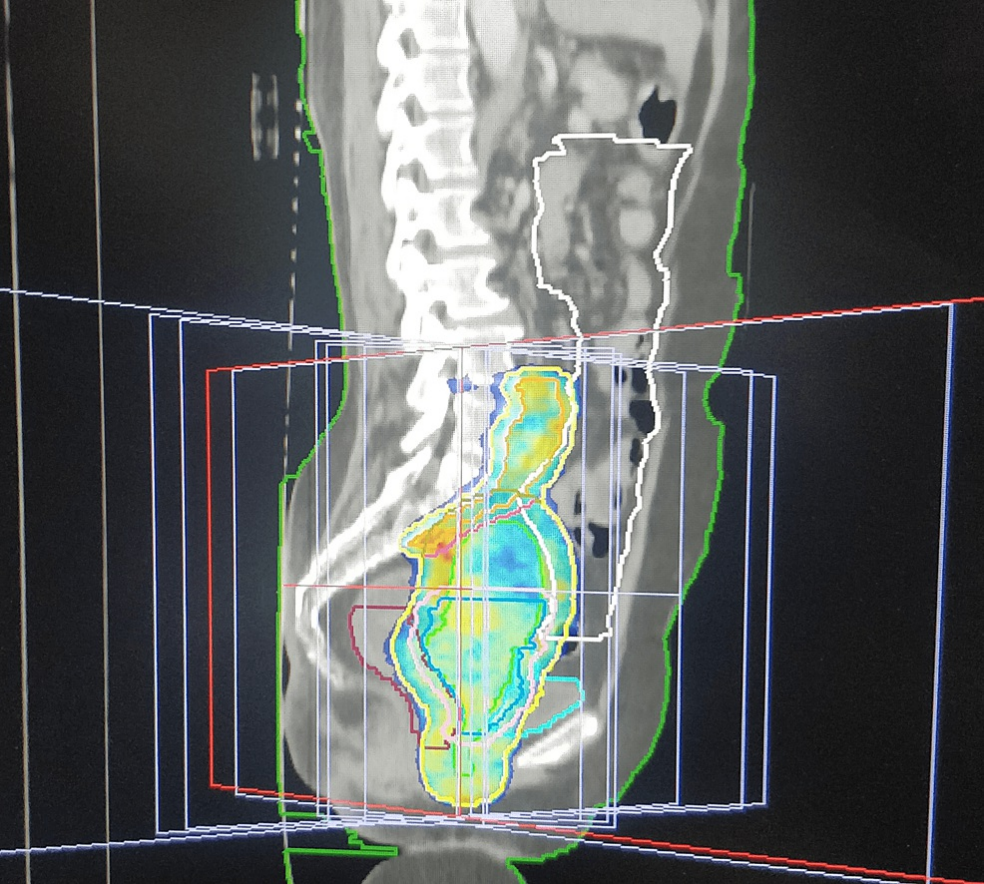
IMRT plan showing the colour wash with 95% isodose coverage (sagittal section) IMRT, intensity-modulated radiotherapy

Then, the patients were randomised into two groups, either 3DCRT or IMRT, by block randomisation. Before the computed tomography (CT) simulation, bowel preparation was done with sodium phosphate proctoclysis enema. The patient was instructed to follow bladder protocol, i.e., to drink 750 mL of water 45 minutes before the scan. (During treatment, the patient is asked to follow the “bladder protocol” before each fraction of radiation.) The patient was placed in a supine position with arms above the head. A four-clamp thermoplastic mask was used as the immobilisation device. A contrast-enhanced CT scan was acquired with a 2.5 mm slice thickness (Siemens SOMATOM Definition AS). Then, contouring of the organs at risk (OAR) (bowel bag, rectum, bladder, and femoral head) was done as per radiation therapy oncology group (RTOG) consensus guidelines [14]. Clinical target volume (CTV), internal target volume (ITV), and planning target volume (PTV) were contoured using the Post Graduate Institute of Medical Education and Research, Chandigarh, India, contouring guidelines [15]. Planning was done in Oncentra 4.2 TPS for 3DCRT and Monaco (version) treatment planning system (TPS) for IMRT (Figures 1-4).

For inverse planning, dose constraints to OARs were bowel bag (V45 < 195 cc), rectum (V40 < 40%), bladder (V40 < 40%), and each femoral head (V45 < 5%). The dose prescriptions in both plans were 50 Gy in 25 fractions over five weeks. All plans were normalised to cover at least 95% of PTV with a 95% prescription dose. For the bowel bag, the mean bowel dose and also V5, V15, V25, V35, V45, and V50 were quantified for each patient. Cone beam CT was used for image guidance in the IMRT arm. It was repeated twice weekly till the completion of the treatment. After external beam radiotherapy (EBRT), intracavitary brachytherapy (ICBT) was given in those cases with an intact cervix, and 7 Gy was prescribed to point A. At the same time, intravaginal brachytherapy (IVBT) was delivered to the post-operative cases. Here, 7 Gy was prescribed at 0.5 cm from the applicator surface. The brachytherapy sessions were weekly once for three weeks for all the patients (both the 3DCRT and IMRT groups).

Acute bowel toxicity was graded as per RTOG toxicity criteria [16]. It was evaluated weekly during treatment, at the end of EBRT, at six weeks, and then 12 weeks after completion of EBRT. Toxicity grading higher than 1 was considered for statistical analysis. The unpaired T-test was used to compare the mean V5/V15/V25/V35/V45/V50 of the bowel bag between the two groups. To compare different factors affecting GI toxicity, like age, stage, treatment technique, chemotherapy, and intention of treatment (radical/adjuvant), Cox regression univariate and multivariate analyses were applied. Data analysis was done by IBM SPSS Statistics, version 24 (IBM Corp., Armonk, NY). For interpreting the results, a p-value of less than 0.05 was considered statistically significant.

## Results

We recruited 24 patients for this study (Table 1). They were randomised into two groups (3DCRT and IMRT), 12 in each arm. All the DVH parameters in IMRT were less than that of the corresponding 3DCRT, and significant differences were found in mean V25, V45, and V50 with statistical significance (Table 2). The incidence of grades 2 and 3 acute GI toxicities was higher in the 3DCRT arm. Grade 2 toxicity was most common in the 3DCRT arm (67%). No patient in the IMRT arm developed grade 3 toxicity (Table 3). In both univariate (p = 0.036) and multivariate (p = 0.028) analyses, it was noted that only the treatment technique was the statistically significant factor (Table 4).

**Table 1:** Baseline characteristics of the study subjects 3DCRT, three-dimensional conformal radiotherapy; IMRT, intensity-modulated radiotherapy; FIGO, The International Federation of Obstetrics and Gynaecology; RT, radiotherapy only; CCRT, concurrent chemoradiotherapy

Group		3DCRT	IMRT
Number of patients		12	12
The age group of patients (in years)	30-39	2	1
	40-49	1	3
	50-59	2	3
	60-69	5	3
	70-79	2	2
FIGO stage	Stage I	0	2
	Stage II	8	6
	Stage III	4	4
	Stage IV	0	0
Histopathology	Squamous cell carcinoma	12	10
	Adenocarcinoma	0	1
	Adenosquamous carcinoma	0	1
Intention of treatment	Radical	11	9
	Adjuvant	1	3
Modality of treatment	RT	2	2
	CCRT	10	10

**Table 2:** Comparison of the mean bowel bag dose in the study groups 3DCRT, three-dimensional conformal radiotherapy; IMRT, intensity-modulated radiotherapy; p-value, probability value; Vx, volume of the bowel bag receiving “x” Gy dose of radiation

Bowel bag dose	Mean V5	Mean V15	Mean V25	Mean V35	Mean V45	Mean V50
3DCRT	61.025	50.325	45.033	22.716	9.387	9.316
IMRT	59.841	48.208	33.400	19.683	6.717	4.050
Significance (p-value)	0.853	0.728	0.047	0.474	0.048	0.029

**Table 3:** The distribution of acute gastrointestinal toxicity in the study groups RTOG, radiation therapy oncology group; 3DCRT, three-dimensional conformal radiotherapy; IMRT, intensity-modulated radiotherapy

RTOG toxicity grading	3DCRT	IMRT
Grade 1	1	6
Grade 2	8	6
Grade 3	3	0

**Table 4:** The factors affecting acute gastrointestinal toxicity (>grade 1) HR, hazard ratio; p-value, probability value; 95% CI, 95% confidence interval

Factors	Univariate analysis			Multivariate analysis		
	HR	p-value	95% CI	HR	p-value	95% CI
Age	1.450	0.473	0.526-3.998	0.832	0.799	0.204-3.404
Stage	0.712	0.530	0.247-2.055	0.657	0.541	0.170-2.528
Treatment technique	3.127	0.036	1.080-9.049	3.578	0.028	1.149-11.134
Chemotherapy	0.725	0.617	0.207-2.548	0.466	0.362	0.090-2.411
Intention (radical/adjuvant)	0.937	0.932	0.209-4.199	0.870	0.880	0.142-5.335

## Discussion

The treatment paradigm of cervical cancer has evolved over the past few decades around surgery, radiotherapy, and chemoradiotherapy; concerning radiotherapy, the treatment techniques have metamorphosed from conventional to conformal and intensity modulation. Pelvis irradiation is associated with a handful of acute and chronic toxicities, the most common being acute GI toxicity. At the outset, we intended to analyse the differences in dose-volume parameters between the two treatment techniques (3DCRT vs IMRT) and to correlate them clinically.

The volume of bowel bag irradiated from low to high doses was numerically higher in 3DCRT than IMRT. The difference in volumes was significant at V25, V45, and V50. These findings were similar to those of Lv et al., Kwak et al., Forrest et al., Naik et al., and Gandhi et al. [17-21].

In this study, 50% of the patients in the IMRT arm developed grade 1, and the remaining 50% developed grade 2 acute GI toxicity. This was observed during the fourth and fifth weeks of treatment. No patients in the IMRT arm developed grade 3 GI toxicity. While in the 3DCRT arm, most patients (66%) developed grade 2 GI toxicity. Unlike the IMRT arm, grade 3 GI toxicity was recorded in the 3DCRT arm. Three patients developed grade 3 GI toxicity during the fifth week of treatment. In Naik et al., grade 1 toxicity was comparable between the 3DCRT and IMRT arms [20]. At the same time, grades 2 and 3 toxicities were significantly less in the IMRT group [20]. Similarly, in Gandhi et al., grades 2 and 3 GI toxicities were substantially less in the IMRT group [21]. In Kwak et al., grade 2 acute GI toxicity is significantly less in the IMRT group [18]. At the same time, grade 1 toxicity is higher in IMRT, which is close to our findings [18].

In our study, we have analysed various factors that affect acute GI toxicity. These factors were the patient’s age, stage of the disease, chemotherapy, treatment technique, and the intention of the treatment. Among these, the treatment technique alone affected acute GI toxicity significantly. This finding resonated with the result of Kwak et al. [18].

We would also like to shed some light on the shortcomings of this study. The sample size in our study is small, and a larger sample size may bring out the correlation between other factors and acute GI toxicity. To evaluate GI toxicity, we have taken RTOG toxicity criteria [16], which are subjective and may be misleading sometimes. Although the effect of concurrent chemotherapy on acute GI toxicity in our study is not statistically significant, it still needs a separate analysis with a larger sample size. Brachytherapy may potentiate the effects of EBRT on GI toxicity, but we haven’t analysed the impact of brachytherapy separately. One of the reasons for this is that the dose of brachytherapy is the same for all patients.

## Conclusions

GI toxicity is frequently encountered during EBRT of cervical cancer. The treatment technique holds a vital role in the development of GI toxicity. IMRT is superior to 3DCRT concerning the dose received by bowel bag, which translated into a reduction in acute GI toxicity. So, with reduced toxicity, IMRT will enhance patients’ quality of life and treatment compliance.
